# Laparoscopic appendectomy in an adult patient with situs inversus totalis

**DOI:** 10.1093/jscr/rjad134

**Published:** 2023-03-14

**Authors:** Trung V Hoang, Huan T Hoang, Hoai T Vo, Vichit Chansomphou

**Affiliations:** Department of Radiology, Thien Hanh Hospital, Buon Ma Thuot, Vietnam; Department of Radiology, Thien Hanh Hospital, Buon Ma Thuot, Vietnam; Department of Radiology, Tam Tri Nha Trang General Hospital, Nha Trang, Vietnam; Department of Radiology, Savannakhet Medical-Diagnostic Center, Kaysone Phomvihane, Laos

**Keywords:** Laparoscopic appendectomy, Left-sided appendicitis, Situs inversus totalis

## Abstract

Left-sided appendicitis is usually caused by situs inversus totalis or midgut malrotation. Clinical and imaging diagnoses have been presented relatively fully in the literature. However, this is a rare condition, and each related case should be further reported to help the day-to-day clinician better investigate and understand. Therefore, in this paper, we present a case of left-sided acute appendicitis in an adult male patient with situs inversus totalis. In addition, we also discuss the laparoscopic technique of the left-sided appendectomy as it is technically more difficult because of the mirror nature of the anatomy.

## INTRODUCTION

There are three types of visceral situs that exist in the body including situs solitus, situs inversus and situs ambiguous. Situs solitus refers to the normal arrangement of organs. Situs inversus refers to a mirror-image arrangement of organs to situs solitus. Situs inversus totalis is a rare congenital disease associated with autosomal recessive inheritance. The condition is a positional anomaly when both the thoracic and abdominal organs develop in the wrong position. The prevalence in the general population is reported to be between 0.001 and 0.01% [1–3].

## CASE REPORT

**Figure 1:**
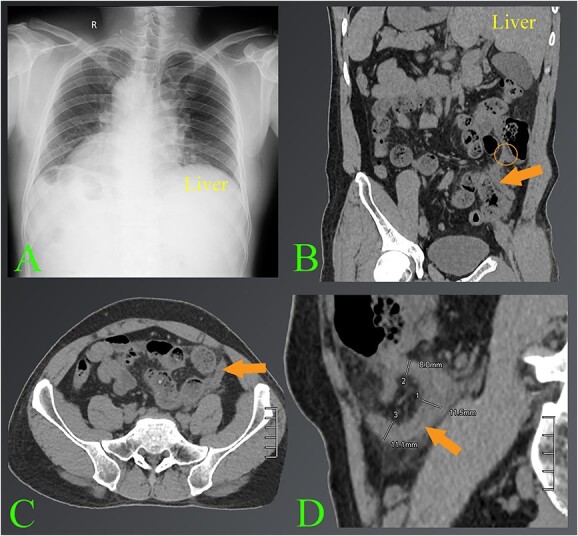
The images of appendicitis in a patient with situs inversus. (**A**) Chest X-ray of the chest shows that the heart is on the right side. (**B**–**D**) The CT images show the left-sided acute appendicitis with surrounding fatty infiltration (arrows). The root of the appendix located high in the left lumbar position is also seen (circle). Note where the liver is located on the left side.

A 42-year-old male admitted to the hospital with dull abdominal pain that had persisted for the previous 10 h. The patient stated that the pain started in the epigastrium and then spread to the left iliac fossa. Accompanying symptoms were nausea and shivering fever. The patient had a known history of organ reversal. The clinician suspected acute appendicitis, ordered routine tests, chest radiograph and abdominal ultrasound immediately afterwards. Complete blood count showed an elevated white blood cell count of about 16 000/mL. The chest radiograph showed the existence of dextrocardia (Fig. 1A). The ultrasound results showed that a sonographer’s attempt was made to find the cause of abdominal pain in the left iliac fossa, but because the patient had a stiff abdomen, the appendix was not detected. The patient underwent a CT scan shortly thereafter. CT images confirmed the diagnosis of the left-sided acute appendicitis with situs inversus totalis (Fig. 1B–D). After carefully assessing the position of the appendix relative to the patient’s condition, a laparoscopic appendectomy was conducted.

**Figure 2:**
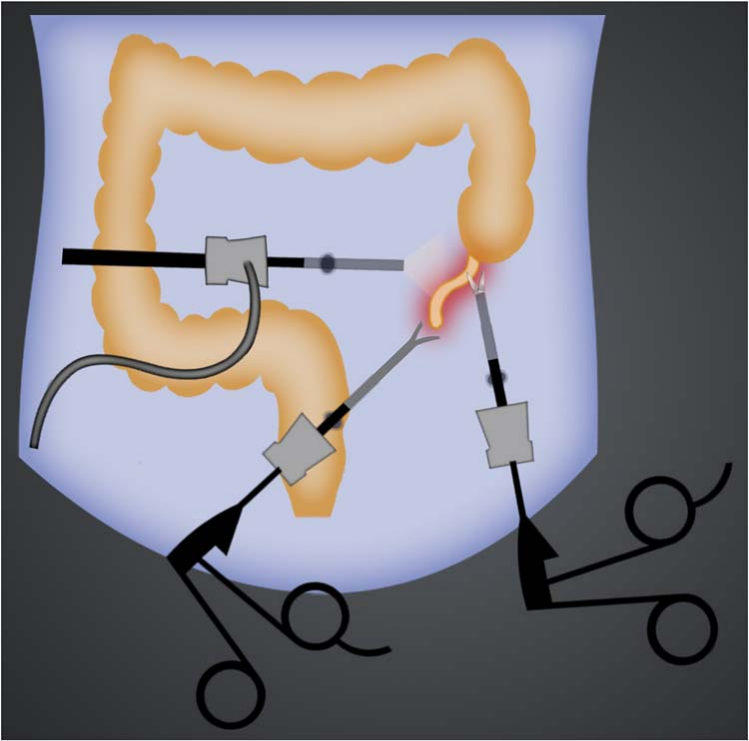
Illustration of port sites of laparoscopic left-sided appendectomy.

The surgeons placed three ports according to experience, as long as they feel most convenient. A 10 mm trocar was placed just below the umbilicus; the other two 5 mm trocars were placed in the left iliac fossa and suprapubic region (Fig. 2). Surgeon and first assistant stand on the patient’s right side and second assistant on the patient’s left side. A video monitor is used on the left side beside the patient’s feet. Once the appendix was identified, it was clamped and raised toward the abdominal wall to expose the mesoappendix. The mesoappendix was then transected with harmonic scalpel. After the appendix was completely released, an endoloop was placed at its root (about 7 mm from the cecum). The appendix was then sectioned with scissors about 5 mm above the endoloop. The appendix specimen was finally removed inside a protective bag through the umbilical port. The surgery was also quite smooth and the operation time was 25 min. The patient was discharged after 3 days without any complications.

## DISCUSSION

Left-sided appendicitis is usually caused by situs inversus totalis or midgut malrotation. The prevalence of appendicitis associated with situs inversus totalis ranged from 0.016 to 0.024%. They are reported to occur more frequently in males than in females (ratio 3/2) and the reported age ranges from 8 to 82 years. There were 62% of patients present with left iliac fossa pain, 14% right iliac fossa pain and 7% bilateral pain. This is explained by the corresponding non-translocation nervous system and referred pain [4, 5].

Laparoscopic appendectomy in situs inversus totalis can be performed with three ports; however, there is no agreed standard portal position. Basically, all positions on the abdominal wall can be inserted trocar according to the basic principle of laparoscopy triangulation, as long as it is most convenient for the surgeon to manipulate. At the same time, port placements are modified based on the patient’s condition and the position of the appendix assessed on imaging studies [3–7].

**Figure 3:**
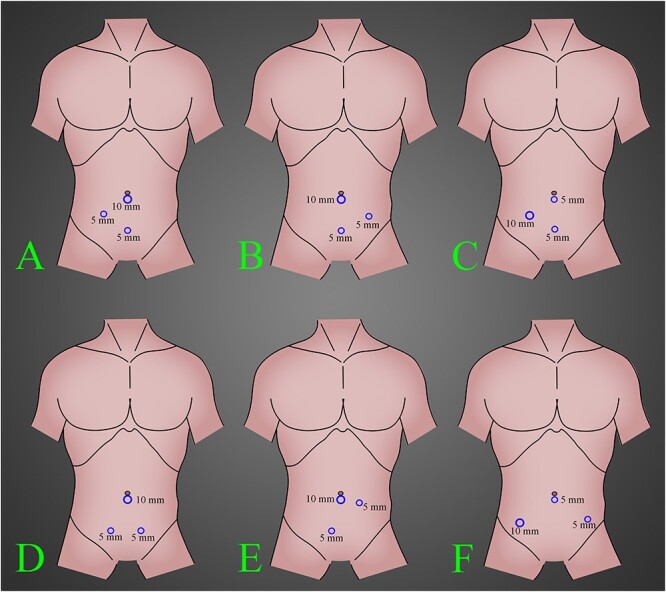
The most common position of the ports in laparoscopic left-side appendectomy according to our knowledge and experience. (**A**) A 10-mm trocar inserted in the umbilical region, a 5-mm trocar inserted in the suprapubic region and a 5-mm trocar inserted in the right lower quadrant region. (**B**) A 10-mm trocar inserted in the umbilical region, a 5-mm trocar inserted in the suprapubic region and a 5-mm trocar inserted in the left lower quadrant region. (**C**) A 10-mm trocar inserted in the right lower quadrant region, a 5-mm trocar inserted in the umbilical region and a 5-mm trocar inserted in the suprapubic region. (**D**) A 10-mm trocar inserted in the umbilical region and two 5-mm trocars inserted in bilateral suprapubic region. (**E**) A 10-mm trocar inserted in the umbilical region, a 5-mm trocar inserted in the right suprapubic region and a 5-mm trocar inserted in the left lower quadrant region, a little to upward. (**F**) A 10-mm trocar inserted in the right lower quadrant region, a 5-mm trocar inserted in the umbilical region and a 5-mm trocar inserted in the left lower quadrant region.

With the appendix on the left side, the surgeon’s preferred port placements include one in the umbilicus, one in the hypogastrium and one in the right iliac fossa (Fig. 3). In our patient, because the appendix root was located high in the left lumbar position, a port placed on the left side can also facilitate surgical manipulation. For this case, a right port can also be placed instead of the left side, which can also easily perform surgery.

These port placements keep the surgeon comfortable and free from arm and wrist fatigue. Handedness has been reported to affect surgical performance in situs inversus. Left-handed or ambidextrous operators can perform the laparoscopic left-side appendectomy more easily. The resection and ligation of the mesoappendix in situs inversus is also more difficult than usual, so harmonic scalpel or electrocautery should be used to perform this procedure instead of scissors and endoloop [8–10].

## CONCLUSIONS

Surgical technique for left appendicitis is not specific. Therefore, the surgeon should base on the position of the appendix on the imaging of each patient to come up with a specific treatment strategy. Surgical techniques should also refer to the literature. However, the technique can be variable and arbitrary, as long as the surgeon finds it convenient and comfortable.

## Data Availability

All available data were included in this published article.
